# Protein expression in the midgut of sugar-fed *Aedes albopictus* females

**DOI:** 10.1186/1756-3305-5-290

**Published:** 2012-12-11

**Authors:** Leonardo Saboia-Vahia, Andre Borges-Veloso, Patricia Cuervo, Magno Junqueira, Camila Mesquita-Rodrigues, Constanca Britto, Gilberto Barbosa Domont, Jose Batista De Jesus

**Affiliations:** 1 Laboratório de Biologia Molecular e Doenças Endêmicas, Instituto Oswaldo Cruz, FIOCRUZ, Rio de Janeiro, Brazil; 2 Laboratório de Pesquisa em Leishmaniose, Instituto Oswaldo Cruz, FIOCRUZ, Rio de Janeiro, Brazil; 3 Unidade de Proteômica, Laboratório de Química de Proteínas, Instituto de Química, Universidade Federal do Rio de Janeiro, Rio de Janeiro, Brazil; 4 Departamento de Engenharia de Biossistemas, Universidade Federal de São João Del Rey, Minas Gerais, Brazil; 5 Laboratório de Bioquímica e Química Proteínas, Departamento de Biologia Celular, Universidade de Brasília, Brasília, Brazil

**Keywords:** *Aedes albopictus*, Culicidae, Midgut, Proteomics, Proteome, Two-dimensional electrophoresis, Mass spectrometry

## Abstract

**Background:**

*Aedes albopictus* is a vector for several fatal arboviruses in tropical and sub-tropical regions of the world. The midgut of the mosquito is the first barrier that pathogens must overcome to establish infection and represents one of the main immunologically active sites of the insect. Nevertheless, little is known about the proteins involved in the defense against pathogens, and even in the processing of food, and the detoxification of metabolites. The identification of proteins exclusively expressed in the midgut is the first step in understanding the complex physiology of this tissue and can provide insight into the mechanisms of pathogen-vector interaction. However, identification of the locally expressed proteins presents a challenge because the *Ae. albopictus* genome has not been sequenced.

**Methods:**

In this study, two-dimensional electrophoresis (2DE) was combined with liquid chromatography in line with tandem mass spectrometry (LC-MS/MS) and data mining to identify the major proteins in the midgut of sugar-fed *Ae. albopictus* females.

**Results:**

Fifty-six proteins were identified by sequence similarity to entries from the *Ae. aegypti* genome. In addition, two hypothetical proteins were experimentally confirmed. According to the gene ontology analysis, the identified proteins were classified into 16 clusters of biological processes. Use of the STRING database to investigate protein functional associations revealed five functional networks among the identified proteins, including a network for carbohydrate and amino acid metabolism, a group associated with ATP production and a network of proteins that interact during detoxification of toxic free radicals, among others. This analysis allowed the assignment of a potential role for proteins with unknown function based on their functional association with other characterized proteins.

**Conclusion:**

Our findings represent the first proteome map of the *Ae. albopictus* midgut and denotes the first steps towards the description of a comprehensive proteome map of this vector. In addition, the data contributes to the functional annotation of *Aedes* spp. genomes using mass spectrometry-based proteomics data combined with complementary gene prediction methods.

## Background

The mosquito *Aedes albopictus* is a vector of fatal arboviruses such as yellow fever, Chikungunya and Dengue, which, according to estimates made by the World Health Organization (WHO) can reach over 50 million cases worldwide each year [1-3]. In Brazil, *Ae. albopictus* has been reported in 21 states with 1,502 municipalities infested [4]. This distribution is consistent with the fact that this species is able to adapt easily to new habitats, particularly those disturbed by man, such as wooded areas occupied by new settlements, and over time, becomes a permanent part of the local fauna [5]. During blood feeding females of *Ae. albopictus* acquire the nutrients necessary for egg maturation and production of yolk proteins [6]. However, during such feeding, females can also be infected with various pathogens, such as Dengue virus, which must cross the midgut epithelial cells to finally reach the salivary glands and ensure their transmission to a new host during the next blood meal. For this reason, many previous studies have focused on the salivary glands with the aim of discovering biomarkers involved in the interaction of tissue cells with the virus or parasite and identifying molecules involved in immune responses at the time of the insect blood meal [7-11]. However, the midgut is the first barrier that pathogens must overcome to establish infection and represents one of the main immunologically active sites of the insect [12]. Thus, many elements including the blood of the vertebrate to be processed, pathogens and the molecules of the vector immune response, among others, converge on the midgut. Nevertheless, little is known about the proteins involved in processing the blood or detoxifying the metabolites from this process. Furthermore, the extent of the proteins involved in defense against pathogens and which are expressed during the mosquito feeding intervals are also unknown. A description of these molecules may help to understand the phenomena that control the development of pathogens and subsequent transmission by the insect. Characterizing the profile of proteins in the midgut of females is one of the first steps to comprehend the complex physiology of this tissue. Proteomic approaches enable the protein profile of a tissue or cell to be fully defined and the proteins expressed under different conditions to be identified. Transcriptomic analyses have made important contributions to understanding the biology of *Aedes* spp., but few proteomic studies have been conducted in this genus. However, proteomic techniques have been used to characterize *Ae. aegypti* subproteomes, such as membrane from larval midgut, adult salivary gland, Malpighian tubules and semen [13-16]. In addition, a proteomic analysis of an *Ae. albopictus* cell line infected by Dengue serotypes 1 and 3 has been reported [17]. In the present study, using an approach that combines two-dimensional electrophoresis, mass spectrometry and data mining, we describe the proteomic map of the midgut from *Ae. albopictus* females.

## Methods

### Chemicals

All reagents were purchased from Sigma (St. Louis, MO, USA) or Merck (São Paulo, SP, Brazil). MilliQ-purified water (Millipore Corp., Bedford, MA, USA) was used to make all of the solutions.

### Insects

Experiments were carried out using female adults (2–5 days old) of *Ae. albopictus* caught in the Brazilian state of Rio de Janeiro and reared in a closed colony in the Laboratório de Transmissores de Hematozoários - Instituto Oswaldo Cruz, FIOCRUZ, Rio de Janeiro. Mosquitoes used in this study had been maintained for near 100 generations in the closed colony. Laboratory maintenance conditions were a temperature of 25±1°C, relative humidity 60±10% and a light:dark photoperiod of 14:10 h. The mosquitoes were maintained on a 10% sucrose diet.

### Gut dissection

The mosquitoes were cold-anesthetized on ice and decapitated. Dissection was performed in cold, sterilized PBS buffer, pH 7.4 (150 mM NaCl, 10 mM Na_2_HPO_4_). The thorax was held with forceps (#5), and the intestine, Malpighian tubules and ovary were dissected by gently pulling at the rectum with another pair of forceps. In order to guarantee the integrity and cleanliness of the midguts, the Malpighian tubules and ovaries were cut out, and the midguts were cut by a longitudinal incision and thoroughly rinsed with PBS to remove the gut contents, including bacteria present as general microbiota. Midguts were then transferred to a microcentrifuge tube. The isolated midguts were digitally imaged using optic microscopy with differential interference contrast.

### Protein extraction

A pool of 50 midguts was lysed in IEF buffer containing 9 M urea, 4% CHAPS, 65 mM dithiothreitol (DTT), and 1% ampholytes (pH 3–10) plus 5 mM PMSF and a cocktail of protease inhibitors. The samples were mechanically lysed using a plastic pestle in combination with 10 cycles of freezing in liquid nitrogen and thawing. The lysate was centrifuged at 10,000 x *g* for 10 min at 4°C, and the proteins in the resulting supernatant were precipitated with methanol:chloroform (3:1). Finally, the pellet was resuspended in IEF buffer (9 M urea, 4% CHAPS, 65 mM dithiothreitol (DTT) and 1% ampholytes, pH 3–10) for 1 h at room temperature. The protein concentration was determined using the 2-D Quant Kit (GE Healthcare).

### 2DE electrophoresis, protein visualization and image analysis

For the first dimension, 100 μg of the protein was diluted to a final volume of 125 μl in a rehydration solution (8 M urea, 2% CHAPS, 65 mM DTT, 1.5% ampholytes, pH 3–10, and 0.001% bromophenol blue). This solution was applied to IEF strips (7 cm, pH 3–10 non-linear; GE Healthcare) and submitted to isoelectric focusing on an Ettan IPGphor 3 (GE Healthcare) at 20°C with a maximum current of 50 μA/strip. The focusing parameters were set as previously described [18]. Reduced (10 mg/mL DTT) and alkylated (25 mg/mL iodoacetamide) proteins were separated on 12% SDS-PAGE gels (30% acrylamide, 0.8% bis-acrylamide) using a vertical system (Bio-Rad) and standard Tris/glycine/SDS buffer. The gels were stained with colloidal Coomassie Brilliant Blue G-250 [19]. Images of the gels were acquired using a GS-800™ calibrated imaging densitometer (Bio-Rad), and image analysis was performed using PDQuest™ software (Bio-Rad). Three gels from three independent gut suspensions were compared. To assign experimental p*I* and Mr coordinates for each single spot, 2DE gels were calibrated using a select set of reliable identification landmarks distributed throughout the entire gel.

### Protein digestion, peptide extraction and analysis by mass spectrometry

The protein spots were manually excised and digested following previously described protocols [18,20]. Briefly, the gel pieces were washed with 50% (v/v) acetonitrile in 25 mM ammonium bicarbonate, dehydrated in 100% acetonitrile, dried and further rehydrated in 50 mM ammonium bicarbonate with 200 ng of trypsin (Promega). The tryptic digestion was performed overnight at 37°C. The peptides were extracted using 0.1% formic acid in 50% (v/v) acetonitrile, concentrated under vacuum and desalted using C18 tips. The eluted peptides were loaded in a nanoLC (Nano-LC Ultra 2D Plus, Eksigent), running a 50 minute gradient. Peptides were fractionated using a gradient from 95% phase A (95% water, 5% Acetonitrile, 0.1% formic acid added to the total) to 40% phase B (85% Acetonitrile, 15% water, 0.1% formic acid added to the total) for 42 min, 40% to 90% phase B during 4 minutes and sustaining 90% phase B for another 4 minutes (total of 50 min at a flow rate of 200 nl/min). Analytical column consisted of ReproSIL Gold c18 3 um diameter beads (Dr Maisch GmbH, Germany) packed in-house inside a 75 um ID silica tip with metalic coating, 12 cm length. The nanoLC was in-line with a hybrid LTQ XL-Orbitrap mass spectrometer, running a Data Dependent Acquisition method, where MS1 was performed on the FTMS at 60000 resolution, followed by CID fragmentation (35 normalized collisional energy) of the 5 most intense ion analyzed on the ITMS with a dynamic exclusion of 90 seconds to prevent re-fragmentation of the same ion. The mass spectra were searched against the non-redundant database of the National Center for Biotechnology (NCBI) using the program Mascot MS/MS ion search (http://www.matrixscience.com/search_form_select.html, Matrix Science, Oxford, UK, free online version). The search parameters were lack of taxonomic restrictions; one tryptic miscleavage; carbamidomethylation of cysteine residues as fixed and oxidation of methionine and acetylation as variable modifications; 10 ppm mass tolerance for the MS mode and 0.6 Da tolerance for its corresponding fragments in MS/MS.

### Bioinformatics analyses of identified proteins

Gene Ontology (GO) annotations of biological process of *Ae. albopictus* proteins were assigned according to those reported in the VectorBase database (http://www.vectorbase.org/) and confirmed at the AmiGO database (http://amigo.geneontology.org/cgi-bin/amigo/search.cgi). The putative function of hypothetical proteins was inferred using the InterProScan Sequence Search (http://www.ebi.ac.uk/Tools/pfa/iprscan/). Identified proteins were also analyzed in terms of putative functional association networks according to the STRING 9.0 Server [21,22] (http://www.string-db.org).

## Results and discussion

### Two-dimensional gel separation and identification of *Ae. albopictus* gut proteins

**Figure 1:**
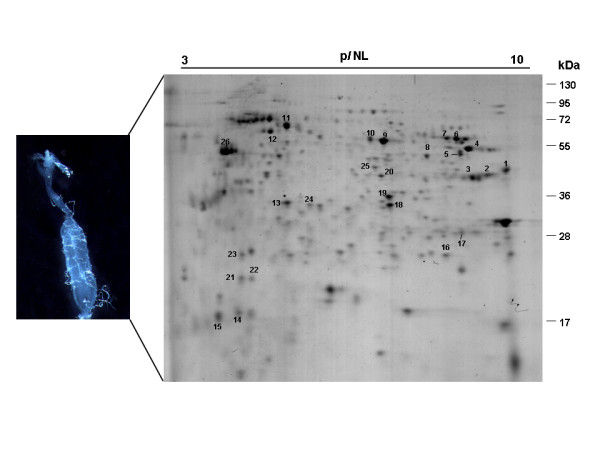
**2DE map of soluble proteins from the midgut of *Ae. albopictus *females.** Proteins were separated in the first dimension across a non-linear pH range of 3–10 NL, and in the second dimension in a 12% SDS-PAGE. The proteins were detected after staining by colloidal Coomassie Brilliant Blue G-250. The protein spots identified by nLC-MS/MS (LTQ-Orbitrap) are numbered, and their identities are provided in Table 1. The numbers on the right side indicate the molecular mass standards expressed in kDa. In the left, optical micrography obtained with camera attached to stereoscope.

The gut proteins were fractionated by 2DE in a non-linear gradient pH 3–10. Image analysis of 2DE gels obtained from three independent experiments showed that the protein spot profiles were highly reproducible in terms of both the total number of protein spots and their relative positions and intensities. Approximately 340 protein spots, distributed in a molecular mass range between ~17 and ~110 kDa and a p*I* range between ~3.5 and ~9.5, were detected in the Coomassie blue G-stained gels (Figure 1). These results agree with previously reported proteomic maps of *Ae. aegypti* midgut where a similar number of spots were resolved [13,14]). In the absence of reported *Ae. albopictus* genome sequences, the proteins were identified based on their similarity with the available *Ae. aegypti* sequences. Automatic search of tandem mass spectra allowed the identification of 56 proteins from 26 protein spots (Table 1, Figure 1). Forty different protein entries were found among the 56 identifications (Tables 1 and 2). The spot numbers in Table 1 correspond to the midgut proteins shown in Figure 1.

**Table 1:** Proteins automatically identified using the Mascot software

**Spot code**	**Protein name**	**NCBI accesion No.**	**VectorBase DB No.**	**Theor/Exp MW**	**Theor/Exp p/**	**Matching pep./Pep. identified by MS/MS**	**Peptide sequence**	**Error ± ppm**	**Protein score**	**Ion score**
1	aspartate aminotransferase [Aedes aegypti]	gi|157128621	AAEL002399	47.6/45.5	9.1/8.2	14(7)	K.KINLGVGAYR.D	4		60
							K.INLGVGAYR.D	4		48
							K.EYSPISGTAEFCK.H	4		50
							R.VGGAFLNGFFPGTK.D	4		77
							K.DIYLPTPSWGNHGPIFR.H	5		16
							R.YYDPSTCGFDFK.G	4		62
							K.GALEDLSK.I	4		52
							K.DGHQIALAQSFAK.N	4		49
							R.AGAFSLICSDKEEAAR.T	4		78
							K.ILIRPMYSNPPIHGAR.L	4		42
							R.LVSEILGDANLK.Q	4		38
							K.LMADR.I	2		23
							R.ISMAGVTTK.N	4		44
							K.NVDYLAEAIHAVTK.	4		80
							K.NVDYLAEAIHAVTK.	5		45
1	ATP synthase alpha subunit mitochondrial [Aedes aegypti]	gi|157131648	AAEL012175	59.5/45.5	9.0/8.2	4(3)	R.VVDALGNAIDGK.G	4	81	34
							K.TALAIDTIINQQR.F	4		57
							K.HALIIYDDLSK.Q	5		66
							K.ALLSQIATDGK.I	4		6
2	d-3-phosphoglycerate dehydrogenase [Aedes aegypti]	gi|157109536	AAEL005336	35.7/32.8	8.0/7.8	12(6)	K.SVLVCDAVDNSCVK.L	1	237	44
							K.LLQDHGIK.V	1		19
							K.LLQDHGIK.V	3		34
							K.GYDAVIVR.S	1		33
							K.ITAEILDAGSGK.L	3		98
							R.AGAGVDNIDIVAATR.N	2		50
							R.KLYSGSELYGK.T	3		88
							K.LYSGSELYGK.T	2		68
							R.MNAFGMR.V	4		32
							K.VVATPHLGASTSEAQVR.V	2		60
							R.VAVEVAEQFIALTGK.S	3		29
							R.VAVEVAEQFIALTGK.S	4		121
2	fructose-bisphosphate aldolase [Aedes aegypti]	gi|157111184	AAEL005766	39.9/32.8	8.4/7.8	5(2)	K.DVQEELAR.I	2	50	37
							K.GILAADESTATCGK.R	3		51
							R.FADIGVENNEDNR.R	3		19
							K.NTPSYQAILENANVLAR.Y	4		28
							K.VTETVLAAVYK.A	0		16
3	fructose-bisphosphate aldolase [Aedes aegypti]	gi|157111184	AAEL005766	39.9/32.8	8.4/7.5	19(14)	K.DVQEELAR.I	3	547	56
							K.GILAADESTATCGK.R	4		112
							R.FADIGVENNEDNR.R	3		79
							R.QLLFTADDR.L	4		36
							R.LQENISGVILFHETLYQK.A	4		53
							K.ADDGTPLAAMLK.K	3		34
							K.GVVDLMGSEGECTTQGLDDLGAR.C	3		74
							K.GVVDLMGSEGECTTQGLDDLGAR.C	3		127
							R.CAQYK.K	4		19
							K.KDGCDFAK.W	-2		51
							K.NTPSYQAILENANVLAR.Y	3		127
							K.NTPSYQAILENANVLAR.Y	4		28
							R.IVPIVEPEILPDGDHDLER.C	-1		36
							R.IVPIVEPEILPDGDHDLER.C	2		75
							K.VTETVLAAVYK.A	3		87
							K.ALNDHHVFLEGTLLKPNMVTAGQSCAK.K	2		69
							K.KPSAQEIALATVLALR.R	4		34
							K.KPSAQEIALATVLALR.R	4		114
							K.AAQDELIK.R	4		68
3	d-3-phosphoglycerate dehydrogenase [Aedes aegypti]	gi|157109536	AAEL005336	35.7/32.8	8.0/7.5	2(2)	K.SVLVCDAVDNSCVK.L	3	149	92
							R.AGAGVDNIDIVAATR.N	3		104
3	Chain A, Crystal Structure Of Aedes Aegypti Alanine Glyoxylate Aminotransferase	gi|116667854	AAEL000640	43.4/32.8	7.1/7.5	1(1)	K.LLMGPGPSNAPQR.V	3	48	48
4	ATP synthase alpha subunit mitochondrial [Aedes aegypti]	gi|157131648	AAEL012175	59.5/53.1	9.0/7.4	18(7)	R.VLSIGDGIAR.V	2	215	41
							R.VLSIGDGIAR.V	3		68
							K.NIQADEMVEFSSGLK.G	5		25
							K.NIQADEMVEFSSGLK.G	0		18
							K.NIQADEMVEFSSGLK.G	1		37
							K.NIQADEMVEFSSGLK.G	3		101
							K.APGIIPR.V	2		41
							R.EPMQTGIK.A	3		28
							K.AVDSLVPIGR.G	3		57
							R.ELIIGDR.Q	3		38
							K.TALAIDTIINQQR.F	2		98
							K.RSTVAQIVK.R	1		38
							K.HALIIYDDLSK.Q	1		21
							R.EAYPGDVFYLHSR.L	3		89
							K.GIRPAINVGLSVSR.V	2		22
							K.LELAQYR.E	1		40
							R.LTELLK.Q	1		25
							K.ITAFER.E	2		31
4	alanine aminotransferase [Aedes aegypti]	gi|157124459	AAEL009872	60.4/53.1	8.6/7.4	2(1)	R.ILVVINPGNPTGQVLSR.D	3	49	49
							R.TTILPQPAK.L	3		42
4	ATP synthase alpha subunit mitochondrial [Aedes aegypti]	gi|157131648	AAEL012175	59.5/53.1	9.0/7.4	7(5)	K.NIQADEMVEFSSGLK.G	-5	224	25
							K.GMALNLEPDNVGVVVFGNDK.L	-5		83
							K.GMALNLEPDNVGVVVFGNDK.L	4		24
							K.GMALNLEPDNVGVVVFGNDK.L	3		41
							K.GMALNLEPDNVGVVVFGNDK.L	3		86
							R.EVAAFAQFGSDLDAATQQLLNR.G	4		149
							R.EVAAFAQFGSDLDAATQQLLNR.G	4		58
5	fructose-bisphosphate aldolase [Aedes aegypti]	gi|157111184	AAEL005766	39.9/51.2	8.4/7.2	5(3)	R.LQENISGVILFHETLYQK.A	4	230	47
							K.GVVDLMGSEGECTTQGLDDLGAR.C	3		78
							K.GVVDLMGSEGECTTQGLDDLGAR.C	4		76
							R.IVPIVEPEILPDGDHDLER.C	4		45
							K.VTETVLAAVYK.A	4		61
6	catalase [Aedes aegypti]	gi|94468602	AAEL013407	48.8/58.4	7.2/7.1	22(14)	K.KTPLAVR.F	3	484	30
							R.FSTVGGESGSADTAR.D	2		110
							K.FYTDDGVWDLVGNNTPIFFIR.D	2		100
							R.DPILFPSFIHTQK.R	2		55
							R.FMNGYGSHTFK.L	1		26
							R.FMNGYGSHTFK.L	1		29
							R.FMNGYGSHTFK.L	3		27
							K.LVNADGKPVYCK.F	2		37
							K.RADELAGADPDYSIR.D	1		36
							K.RADELAGADPDYSIR.D	3		61
							R.ADELAGADPDYSIR.D	2		87
							K.GEYPSWTLK.I	0		31
							K.IQVMTFEQAEK.L	4		83
							K.IWPQAEFPLIPVGR.M	2		64
							K.NYFAEVEQIAFDPSSMVPGIEASPDK.M	3		75
							R.LFAYTDTHR.H	4		70
							R.LGANYTQLPVNCPYR.V	1		89
							K.HSVSGDIDR.F	3		48
							K.HSVSGDIDRFESGDEENFAQASVFYR.R	2		33
							R.FESGDEENFAQASVFYR.R	5		74
							R.MISNLVNHMSNASPFIQER.A	2		60
							K.NFAEVDADFGR.Q	1		75
6	catalase [Aedes aegypti]	gi|157135803	AAEL013407	57.1/58.4	7.7/7.1	8(4)	R.NPAENQLNLFK.E	1	196	60
							K.KTPLAVR.F	3		30
							R.FSTVGGESGSADTAR.D	2		110
							K.FYTDDGVWDLVGNNTPIFFIR.D	2		100
							R.DPILFPSFIHTQK.R	2		55
							R.FMNGYGSHTFK.L	1		26
							R.FMNGYGSHTFK.L	1		29
							R.FMNGYGSHTFK.L	3		27
6	ATP synthase alpha subunit mitochondrial [Aedes aegypti]	gi|157131648	AAEL012175	59.5/58.4	9.0/7.1	4(4)	K.NIQADEMVEFSSGLK.G	3	146	86
							K.TALAIDTIINQQR.F	2		60
							R.EVAAFAQFGSDLDAATQQLLNR.G	1		75
							R.EVAAFAQFGSDLDAATQQLLNR.G	13		82
6	catalase [Aedes aegypti]	gi|94468602	AAEL013407	48.8/58.4	7.2/7.1	22(14)	K.KTPLAVR.F	3	484	30
							R.FSTVGGESGSADTAR.D	2		110
							K.FYTDDGVWDLVGNNTPIFFIR.D	2		100
							R.DPILFPSFIHTQK.R	2		55
							R.FMNGYGSHTFK.L	1		26
							R.FMNGYGSHTFK.L	1		29
							R.FMNGYGSHTFK.L	3		27
							K.LVNADGKPVYCK.F	2		37
							K.RADELAGADPDYSIR.D	1		36
							K.RADELAGADPDYSIR.D	3		61
							R.ADELAGADPDYSIR.D	2		87
							K.GEYPSWTLK.I	0		31
							K.IQVMTFEQAEK.L	4		83
							K.IWPQAEFPLIPVGR.M	2		64
							K.NYFAEVEQIAFDPSSMVPGIEASPDK.M	3		75
							R.LFAYTDTHR.H	4		70
							R.LGANYTQLPVNCPYR.V	1		89
							K.HSVSGDIDR.F	3		48
							K.HSVSGDIDRFESGDEENFAQASVFYR.R	2		33
							R.FESGDEENFAQASVFYR.R	5		74
							R.MISNLVNHMSNASPFIQER.A	2		60
							K.NFAEVDADFGR.Q	1		75
6	catalase [Aedes aegypti]	gi|157135803	AAEL013407	57.1/58.4	7.7/7.1	8(4)	R.NPAENQLNLFK.E	1	196	60
							K.KTPLAVR.F	3		30
							R.FSTVGGESGSADTAR.D	2		110
							K.FYTDDGVWDLVGNNTPIFFIR.D	2		100
							R.DPILFPSFIHTQK.R	2		55
							R.FMNGYGSHTFK.L	1		26
							R.FMNGYGSHTFK.L	1		29
							R.FMNGYGSHTFK.L	3		27
6	ATP synthase alpha subunit mitochondrial [Aedes aegypti]	gi|157131648	AAEL012175	59.5/58.4	9.0/7.1	4(4)	K.NIQADEMVEFSSGLK.G	3	146	86
							K.TALAIDTIINQQR.F	2		60
							R.EVAAFAQFGSDLDAATQQLLNR.G	1		75
							R.EVAAFAQFGSDLDAATQQLLNR.G	13		82
6	glutamate dehydrogenase [Aedes aegypti]	gi|157126232	AAEL010464	61.6/58.4	8.3/7.1	5(2)	R.DSGDYEMITGYR.A	0	52	43
							R.GVFHGLDNFIK.E	1		27
							R.AGATCIGIIEHDGSIFNPQGIDPK.A	1		21
							K.DIVHSGLDYTMER.S	3		24
							K.YNLGLDLR.S	2		57
7	catalase [Aedes aegypti]	gi|94468602	AAEL013407	48.8/58.4	7.2/7.0	22(16)	K.KTPLAVR.F	0	461	37
							R.FSTVGGESGSADTAR.D	2		99
							K.FYTDDGVWDLVGNNTPIFFIR.D	1		66
							K.FYTDDGVWDLVGNNTPIFFIR.D	2		92
							R.DPILFPSFIHTQK.R	2		25
							R.DPILFPSFIHTQK.R	2		62
							R.FMNGYGSHTFK.L	1		26
							R.FMNGYGSHTFK.L	3		38
							K.LVNADGKPVYCK.F	0		45
							K.RADELAGADPDYSIR.D	-1		56
							K.RADELAGADPDYSIR.D	1		40
							R.ADELAGADPDYSIR.D	2		86
							K.IQVMTFEQAEK.L	-1		74
							K.IWPQAEFPLIPVGR.M	2		40
							K.NYFAEVEQIAFDPSSMVPGIEASPDK.M	3		37
							R.LFAYTDTHR.H	0		70
							R.LFAYTDTHR.H	1		52
							R.LGANYTQLPVNCPYR.V	1		80
							K.HSVSGDIDR.F	2		56
							R.FESGDEENFAQASVFYR.R	1		111
							R.MISNLVNHMSNASPFIQER.A	-		28
							K.NFAEVDADFGR.Q	2		73
7	catalase [Aedes aegypti]	gi|157135803	AAEL013407	57.1/58.4	7.7/7.0	9(6)	R.NPAENQLNLFK.E	-9	221	59
							K.KTPLAVR.F	0		37
							R.FSTVGGESGSADTAR.D	2		99
							K.FYTDDGVWDLVGNNTPIFFIR.D	1		66
							K.FYTDDGVWDLVGNNTPIFFIR.D	2		92
							R.DPILFPSFIHTQK.R	2		25
							R.DPILFPSFIHTQK.R	2		62
							R.FMNGYGSHTFK.L	1		26
							R.FMNGYGSHTFK.L	3		38
7	pyruvate kinase [Aedes aegypti]	gi|157107887	AAEL014913	58.0/58.4	7.1/7.0	12(4)	R.LSGIICTIGPASVAPEMLEK.M	0	139	59
							K.MMATGMNIAR.L	2		18
							K.IENQQGMQNLDAIIAASDGIMVAR.G	2		67
							R.AGKPVICATQMLESMIK.K	0		16
							R.AEISDVANAIIDGADCVMLSGETAK.G	3		27
							R.AEISDVANAIIDGADCVMLSGETAK.G	3		51
							K.EAEAALWHR.N	1		19
							R.AAAVIVITTSGR.S	1		65
							R.QCHLYR.G	1		9
							R.GILPVIYEQQALEDWLK.D	1|		74
							R.VQYGMDFGK.E	2		18
							R.GFLKPGNPVVVVTGWK.Q	3		22
7	leucine aminopeptidase [Aedes aegypti]	gi|157121025	AAEL001649	56.6/58.4	6.5/7.0	5(3)	R.ECLFASGCAVAR.A	-1	134	46
							K.AAADPPALAVLSYEPEGATETVAWVGK.G	3		50
							K.AAADPPALAVLSYEPEGATETVAWVGK.G	3		13
							K.VILDMATLTGAQGIATGK.Y	0		113
							K.YHGAILTNSGSWENK.A	-5		42
7	glutamate dehydrogenase [Aedes aegypti]	gi|157126232	AAEL010464	61.6/58.4	8.3/7.0	12(6)	R.FFDMVEYFFHR.A	1	113	43
							R.DSGDYEMITGYR.A	1		58
							K.GFIGPGIDVPAPDMGTGER.E	3		46
							R.GVFHGLDNFIK.E	3		36
							K.EANYMAMIGTTPGWGGK.T	-3		32
							R.AGATCIGIIEHDGSIFNPQGIDPK.A	2		23
							K.IIAEAANGPTTPAADK.I	3		83
							K.IPVTPSEAFQK.R	1		28
							K.DIVHSGLDYTMER.S	2		34
							K.YNLGLDLR.S	0		44
							R.SAAYVNSIEK.I	2		35
							K.IFQTYR.D	1		21
8	enolase [Aedes aegypti]	gi|157121051	AAEL001668	46.8/51.2	6.2/6.7	3(2)	R.GNPTVEVDLVTDLGLFR.A	2	56	17
							K.EALNLIQDAIAK.A	0		54
							K.DFPIVSIEDPFDQDHWDAWAK.M	β		42
9	dihydrolipoamide dehydrogenase [Aedes aegypti]	gi|157114623	AAEL006928	54.1/55.0	6.3/6.2	7(5)	K.NDTLGGTCLNVGCIPSK.A	1	286	120
							R.LDLDVLMDQK.T	-6		64
							K.MADGSEEVVNAK.N	2		118
							K.MADGSEEVVNAK.N	1		82
							K.FLLGTK.V	1		31
							R.RPYTEGLGLENVGIVK.D	3		100
							R.VCHAHPTCAEALR.E	0		39
9	thioredoxin reductase [Aedes aegypti]	gi|157132842	AAEL002886	54.4/55	6.2/6.2	10(8)	K.DAHNVVAVMK.N	0	255	57
							K.DAHNVVAVMK.N	0		19
							K.GFGYDATVMVR.S	0		75
							R.GFDQQMATMVGDAMVEK.G	1		74
							R.GFDQQMATMVGDAMVEK.G	4		61
							K.LDQAGVVTAEGGK.S	2		101
							K.RPELTPVAIHAGR.L	-3		85
							R.YCYLK.A	1		19
							K.AVALLEGDQK.V	-1		82
							K.SSGLDPTPATCCS.	4		54
9	succinyl-coa:3-ketoacidcoenzyme a transferase [Aedes aegypti]	gi|157128446	AAEL011137	49.8/55	6.1/6.2	1(1)	K.AHIADEAGNLIFNK.S	0	62	62
10	thioredoxin reductase [Aedes aegypti]	gi|157132842	AAEL002886	54.4/58.4	6.2/6.0	14(12)	M.APINQENFDYDLVVIGGGSGGLACAK.E	0	389	83
							M.APINQENFDYDLVVIGGGSGGLACAK.E	3		86
							K.LMHQASLLGEAIHDAQPYGWK.F	1		40
							K.LMHQASLLGEAIHDAQPYGWK.F	0		45
							K.LMHQASLLGEAIHDAQPYGWK.F	2		73
							K.VEYVNGLGYFK.D	-2		67
							K.DAHNVVAVMK.N	0		29
							K.DAHNVVAVMK.N	2		53
							R.GFDQQMATMVGDAMVEK.G	-1		98
							R.GFDQQMATMVGDAMVEK.G	3		25
							K.LDQAGVVTAEGGK.S	2		103
							K.AVALLEGDQK.V	-1		78
							K.NTVGIHPTVAEEFTR.L	-2		65
							K.SSGLDPTPATCCS.-	3		52
10	dihydrolipoamide dehydrogenase [Aedes aegypti]	gi|157114623	AAEL006928	54.1/58.4	6.3/6.0	7(7)	K.NDTLGGTCLNVGCIPSK.A	1	285	127
							K.ALLNNSHYYHMAHSGDLASR.G	-4		51
							K.MADGSEEVVNAK.N	2		95
							K.MADGSEEVVNAK.N	2		89
							R.RPYTEGLGLENVGIVK.D	0		64
							R.VCHAHPTCAEALR.E	3		32
							R.VCHAHPTCAEALR.E	4		58
10	thioredoxin reductase [Aedes aegypti]	gi|157132842	AAEL002886	54.4/58.4	6.2/6.0	5(5)	K.TLVVGAGYIGLECAGFLK.G	1	244	115
							R.SDDGTEGSDVYDTVLFAIGR.T	3		115
							K.SDKLDVDSFETTNVPNIFAVGDVLYK.R	1		80
							K.LDVDSFETTNVPNIFAVGDVLYK.R	2		43
							K.VLGLHFLGPVAGEVIQGFAAALK.S	1		40
10	dihydrolipoamide dehydrogenase [Aedes aegypti]	gi|157114623	AAEL006928	54.1/58.4	6.3/6.0	3(2)	R.LGAEVTAIEFLSSIGGAGIDQEVSK.S	0	56	57
							R.VPVNSVFQTIVPSIYAIGDCIHGPMLAHK.A	4		43
							R.VLGVHIIGPAAGELINEAVLAMEYGASAEDVAR.V	0		36
11	transferrin [Aedes aegypti]	gi|157129886	AAEL011641	87.1/66.9	4.9/5.4	16(11)	K.FSEQCLQLQR.G	-1	411	53
							R.GNPEVVCVTVQDSIECAQR.I	0		121
							R.EVVDFR.S	1		37
							R.SVVVVSSQHQGGVDGLR.N	1		77
							K.KFCHPGLHYGR.Q	-4		26
							K.KFCHPGLHYGR.Q	1		46
							K.FCHPGLHYGR.Q	1		35
							K.FCHPGLHYGR.Q	2		30
							R.QLPIPSDLCQTTSR.W	0		98
							R.WCTTSPEEK.D	0		31
							R.TAALTTGIFPTIECVDPTTSR.M	1		107
							R.ADFTGIDSNFGYLAR.H	2		96
							K.YSSVVVLVR.A	0		65
							R.FENLR.N	2		26
							R.GIFDQHECDYGR.L	-1		31
							R.GIFDQHECDYGR.L	0		74
12	chaperonin-60kD, ch60 [Aedes aegypti]	gi|157129785	AAEL011584	61.1/61.8	5.4/5.3	10(6)	K.LVQDVANNTNEEAGDGTTTATVLAR.A	-2	204	69
							K.LVQDVANNTNEEAGDGTTTATVLAR.A	0		48
							K.GANPVEIR.R	0		24
							R.RGVMLAVDAVK.D	1		40
							K.APGFGDNR.K	2		34
							K.LEDVQMSDLGQVGEITITK.D	-1		125
							K.IGGSSEVEVNEK.K	0		85
							K.TLENLK.G	1		31
							R.ALHQPCTQIAK.N	1		43
							K.NAGVDGSVVVAK.V	-1		79
12	chaperonin-60kD, ch60 [Aedes aegypti]	gi|157129785	AAEL011584	61.1/61.8	5.4/5.3	16(9)	R.ALMLQGVDVLADAVAVTMGPK.G	0	431	76
							R.ALMLQGVDVLADAVAVTMGPK.G	2		8
							R.ALMLQGVDVLADAVAVTMGPK.G	2		30
							R.ALMLQGVDVLADAVAVTMGPK.G	4		34
							R.ALMLQGVDVLADAVAVTMGPK.G	-4		77
							R.ALMLQGVDVLADAVAVTMGPK.G	4		103
							K.TLHDELEIIEGMK.F	-1		21
							R.KPLVIIAEDVDGEALSTLVVNR.L	-6		43
							R.KPLVIIAEDVDGEALSTLVVNR.L	-1		40
							R.KPLVIIAEDVDGEALSTLVVNR.L	2		7
							R.KPLVIIAEDVDGEALSTLVVNR.L	3		111
							K.STLSDMAISTGGIVFGDDANLVK.L	2		99
							K.STLSDMAISTGGIVFGDDANLVK.L	5		52
							K.STLSDMAISTGGIVFGDDANLVK.L	1		104
							K.STLSDMAISTGGIVFGDDANLVK.L	2		47
							K.LEDVQMSDLGQVGEITITK.D	1		114
12	dihydrolipoamide dehydrogenase [Aedes aegypti]	gi|157114623	AAEL006928	54.1/61.8	6.3/5.3	1(1)	R.MGLIGAGVIGLELGSVWGR.L	2	103	103
13	anterior fat body protein [Aedes aegypti]	gi|157110227	AAEL000757	39.7/34.4	6.1/5.4	2(1)	K.FVEQFDK.V	3	73	44
							K.FYYADTGAYDVK.V	3		73
14	cytochrome b5, putative [Aedes aegypti]	gi|157108002	AAEL014935	11.7/18.1	4.9/5.0	7(4)	K.TFSLAEIK.A	0	94	25
							K.DATEAFEDVGHSTDAR.E	0		77
							K.DATEAFEDVGHSTDAR.E	0		38
							K.VGELIESER.K	0		62
							R.KQVPVK.K	1		23
							K.KEPDWSTEQK.D	1		35
							K.EPDWSTEQK.D	0		14
14	electron transport oxidoreductase [Aedes aegypti]	gi|157137180	AAEL013739	34.4/18.1	8.4/5.0	2(1)	K.SDLTEFVSQELTK.S	4	66	66
							K.SDRPSLTAAK.I	-17		12
15	60S acidic ribosomal protein P2 [Aedes aegypti]	gi|157105859	AAEL014583	11.3/17.0	4.5/4.8	3(2)	K.ILSSVGIEADSTR.V	3	105	87
							K.SVEELIASGR.E	4		49
							K.LSSMPAGGAAPAAGAGAAAGGAAAAPAEEK.K	4		62
15	hypothetical protein AaeL_AAEL005270 [Aedes aegypti]	gi|157109287	AAEL005270	15.5/17.0	4.8/4.8	4(3)	K.WVWTNAHGPYPPNMVSGGQDSDGALLYVGR.A	3	103	77
							K.ELIWDSATGGNIPPDAVVGGNTADGEPLYIGR.A	4		11
							R.AYHEGSQTIGK.V	-19		26
							R.AYHEGSQTIGK.V	6		54
							R.SHGCCYIPYGGAEVSVPTYDVLCER.-	3		46
15	arp2/3 complex 16 kd subunit (P16-arc) [Aedes aegypti]	gi|157120584	AAEL009059	16.9/17.0	4.7/4.8	2(1)	K.NTSSSAFR.K	4	51	13
							K.VVLQNAPLLCK.N	3		51
16	superoxide dismutase, Mn [Aedes aegypti]	gi|157107594	AAEL004823	24.6/25.8	8.3/6.9	5(2)	K.HTLPDLPYDFGALEPVICR.E	5	153	35
							R.EIMEVHHQK.H	3		45
							K.HHNAYVTNLNAAEEQLAEAVAK.K	3		122
							R.SDPSAELKK.L	-4		30
							K.NLRPNYVDAIWDVVNWK.D	7		83
16	phosphatidylethanolaminebinding protein [Aedes aegypti]	gi|94469304	AAEL011268	25.1/25.8	5.8/6.9	2(1)	R.IAFVGSGPPQGSGLHR.Y	1	85	27
							K.YNLGELVAGNFYR.A	5		85
17	cyclohex-1-ene-1-carboxyl-CoA hydratase, putative [Aedes aegypti]	gi|157104013	AAEL003993	31.9/29.6	8.7/7.3	5(4)	K.NVALITLNRPK.A	2	142	58
							K.CITGNFLNNWTSVAK.A	4		28
							K.ISTFSPLIVQLCK.E	4		59
							K.EAVNTAYETTLNEGLK.F	4		108
							K.DRLEGMTAFVEK.R	3		38
17	3-hydroxyisobutyrate dehydrogenase [Aedes aegypti]	gi|157137993	AAEL013904	34.1/29.6	8.7/7.3	2(2)	K.GAVTYDNVSELAK.A	3	85	67
							K.VFSDIINASTGR.S	2		69
18	pyridoxine kinase [Aedes aegypti]	gi|157123752	AAEL009601	34.0/26.9	6.5/6.3	3(3)	R.VLSIQSHVVHGYVGNK.S	-6	115	63
							R.VLSIQSHVVHGYVGNK.S	3		57
							K.FDMCATLER.T Oxidation (M)	3		76
19	malate dehydrogenase [Aedes aegypti]	gi|157116681	gi|157116681 AAEL007707	36.4/25.8	6.0/6.3	9(5)	K.GDVFGPNQR.L	2	204	28
							K.VLVVGNPANTNALVCSHYAPSIPK.E	6		33
							K.ENFTAMTR.L	1		23
							K.ENFTAMTR.L Oxidation (M)	2		41
							K.DGEYVSMGVVSDGSYGTPK.D	4		118
							K.DGEYVSMGVVSDGSYGTPK.D Oxidation (M)	4		97
							K.IVQGLSVDDFAR.G	4		30
							K.ELLEEKEEAMSVCASD.-	4		42
							K.ELLEEKEEAMSVCASD.- Oxidation (M)	4		52
19	fructose-bisphosphate aldolase [Aedes aegypti]	gi|157111184	AAEL005766	39.9/25.8	8.4/6.3	4(2)	R.FADIGVENNEDNR.R	3	68	14
							R.LQENISGVILFHETLYQK.A	4		53
							K.NTPSYQAILENANVLAR.Y	6		63
							R.IVPIVEPEILPDGDHDLER.C	3		18
20	cystathionine beta-lyase [Aedes aegypti]	gi|157104405	AAEL004059	44.3/18.1	6.0/5.1	11(7)	R.AVVPPISMSTTFK.Q	3	194	44
							K.QFGPAQHAGYEYGR.S	3		21
							K.MNIEVDFVDCTDLAK.V	5		49
							K.MNIEVDFVDCTDLAK.V Oxidation (M)	4		83
							K.VEAAVKPNTK.L	-5		48
							K.LFWIETPTNPLLK.V	5		65
							K.FLQNAAGIVPSPFDCYLVNR.S	5		66
							K.FLQNAAGIVPSPFDCYLVNR.S	6		34
							R.VLHPGLPSHPQHELAK.K	2		29
							R.VLHPGLPSHPQHELAK.K	3		18
							R.ISVGLEDADDLIVDLK.Q	6		110
20	hypothetical protein AaeL_AAEL015064 [Aedes aegypti]	gi|157108923	AAEL015064	10.9/18.1	5.6/5.1	3(2)	K.GSESEHINLK.V	2	81	52
							R.KLMNAYCDR.A Oxidation (M)	2		37
							R.KLMNAYCDR.A Oxidation (M)	-6		77
21	mitochondrial F0 ATP synthase D chain, putative [Aedes aegypti]	gi|157106010	AAEL004423	19.6/22.5	5.2/5.1	3(3)	R.IAQSSVNWAALAER.V	3	133	92
							R.IADYQSQIAALK.A	3		90
							K.ALLPFDQMTMEDYR.D 2 Oxidation (M)	3		48
21	cystathionine beta-lyase [Aedes aegypti]	gi|157104405	AAEL004059	44.3/22.5	6.02/5.1	1(1)	R.ISVGLEDADDLIVDLK.Q	6	118	118
22	lactoylglutathione lyase [Aedes aegypti]	gi|157104950	AAEL014393	20.7/22.5	5.3/5.1	4(1)	K.DFLFQQTMYR.I	2	90	32
							K.ATLELTHNWGTESDPDQK.Y	3		35
							R.GYGHIGIMVPDVEK.A Oxidation (M)	1		37
							K.DPDGYWIEIFNASK.V	6		90
23	glutathione s-transferase [Aedes aegypti]	gi|157130284	AAEL011741	27.0/25.8	5.2/5.1	7(6)	R.FLLSYGNLPFDDIR.I	5	179	90
							R.EEWPALKPTMPMGQMPVLSVDGK.K	4		54
							K.KVHQSVAMSR.Y Oxidation (M)	3		19
							K.QVGLAGADDWENLMIDTVVDTINDFR.L	8		64
							K.IAVVSYEPDDDVKEK.K	2		49
							K.LVTLNSEVIPFYLEK.L	6		49
							R.VVDNVTSIDSIK.A	3		82
23	rho guanine dissociation factor [Aedes aegypti]	gi|157134192	AAEL012996	23.0/25.8	5.0/5.1	1(1)	K.EALLGEAQSEK.I	3	67	67
24	anterior fat body protein [Aedes aegypti]	gi|157110227	AAEL000757	39.7/35.2	6.1/5.5	2(1)	K.FVEQFDK.V	1	83	49
							K.FYYADTGAYDVK.V	5		83
25	aliphatic nitrilase, putative [Aedes aegypti]	gi|157125650	AAEL010284	43.9/47.4	6.0/5.9	6(2)	R.HIPPEELR.E	-6	50	13
							R.HIPPEELR.E	3		34
							K.QYNMVIISPILER.D Oxidation (M)	7		51
							R.KNHIPR.V	1		36
							K.NHIPR.V	1		36
							K.DFWGFPMTQR.L Oxidation (M)	4		16
26	ATP synthase beta subunit [Aedes aegypti]	gi|157132308	AAEL002827	53.8/53.1	5.0/4.9	34(27)	R.LVLEVAQHLGENTVR.T	1	693	47
							R.TIAMDGTEGLVR.G	1		65
							R.TIAMDGTEGLVR.G Oxidation (M)	1		88
							R.VLDTGSPIR.I	2		56
							R.IPVGAETLGR.I	0		63
							R.IINVIGEPIDER.G	2		80
							R.GPIETNLSAPIHAEAPEFIDMSVEQEILVTGIK.V	8		17
							R.GPIETNLSAPIHAEAPEFIDMSVEQEILVTGIK.V Oxidation (M)	5		51
							K.VVDLLAPYAK.G	1		36
							K.IGLFGGAGVGK.T	1		20
							K.TVLIMELINNVAK.A	6		69
							R.EGNDLYNEMIEGGVISLK.D	2		80
							R.EGNDLYNEMIEGGVISLK.D	4		95
							R.EGNDLYNEMIEGGVISLK.D Oxidation (M)	5		100
							K.VALVYGQMNEPPGAR.A Oxidation (M)	3		89
							R.VALTGLTVAEYFR.D	4		75
							R.DQEGQDVLLFIDNIFR.F	6		60
							R.FTQAGSEVSALLGR.I	3		90
							R.IPSAVGYQPTLATDMGSMQER.I	4		73
							R.IPSAVGYQPTLATDMGSMQER.I Oxidation (M)	3		93
							R.IPSAVGYQPTLATDMGSMQER.I Oxidation (M)	5		86
							R.IPSAVGYQPTLATDMGSMQER.I 2 Oxidation (M)	4		78
							R.AIAELGIYPAVDPLDSTSR.I	3		30
							R.AIAELGIYPAVDPLDSTSR.I	5		54
							R.IMDPNIIGAEHYNIAR.G Oxidation (M)	2		20
							K.ILQDYK.S	0		35
							K.SLQDIIAILGMDELSEEDK.L Oxidation (M)	10		75
							K.SLQDIIAILGMDELSEEDKLTVAR.A	6		84
							K.SLQDIIAILGMDELSEEDKLTVAR.A Oxidation (M)	5		89
							K.SLQDIIAILGMDELSEEDKLTVAR.A Oxidation (M)	7		67
							R.FLSQPFQVAEVFTGHAGK.L	3		42
							R.FLSQPFQVAEVFTGHAGK.L	5		94
							K.ILNGELDHLPEVAFYMVGPIEEVVEK.A	6		55
26	protein disulfide isomerase [Aedes aegypti]	gi|157107430	AAEL000641	56.2/53.1	4.9/4.9	9(4)	K.EEDGVLVLTK.D	2		11
							K.AVFDGEYTEEALK.K	3		66
							K.AVFDGEYTEEALKK.F	4		62
							K.NHLLFFISK.E	2		32
							K.ILFVTIDADQEDHQR.I	4		75
							K.KDEVPSMR.I	1		29
							K.KDEVPSMR.I Oxidation (M)	3		39
							R.IIHLEEDMAK.Y Oxidation (M	2		22
							K.MDATANELEHTK.I	0		28

**Table 2:** Biological process categories of the identified proteins

**Categories**^**a**^	**Number of identified spots**
**Amino acid Metabolism**	**10**
Aspartate aminotransferase	1
d-3-phosphoglycerate dehydrogenase	2
Alanine aminotransferase	1
Glutamate dehydrogenase	2
3-hydroxyisobutyrate dehydrogenase	1
Cystathionine beta-lyase	2
Chain A, Alanine Glyoxylate aminotransferase	1
**Response to oxidative stress**	**5**
Catalase	4
Superoxide dismutase, Mn	1
**Cell redox homeostasis**	**8**
Thioredoxin reductase	3
Protein disulfide isomerase	1
Dihydrolipoamide dehydrogenase	4
**Glycolysis**	**6**
Fructose-bisphosphate aldolase	4
Pyruvate kinase	1
Enolase	1
**Proteolysis**	**1**
Leucine aminopeptidase	1
**ATP biosynthetic process**	**6**
ATP synthase alpha subunit mitochondrial	4
Mitochondrial F0 ATP synthase D chain, putative	1
ATP synthase beta subunit	1
**Electron carrier activity**	**2**
Cytochrome b5, putative	1
Electron transport oxidoreductase	1
**Protein folding/chaperons**	**2**
Chaperonin-60kD, ch60	2
**Carbohydrate metabolic process**	**1**
Malate dehydrogenase	1
**Nitrogen compound metabolic process**	**1**
Aliphatic nitrilase, putative	1
**Actin filament polymerization/Cytoskeleton associated**	**1**
arp2/3 complex 16 kd subunit (P16-arc)	1
**Translational elongation / DNA synthesis**	**1**
60S acidic ribosomal protein P2	1
**Ketone body catabolic process**	**1**
Succinyl-coa:3-ketoacid-coenzyme a transferase	1
**Iron ion Transport**	**1**
Transferrin	1
**Phosphorylation**	**1**
Pyridoxine kinase	1
**Unknown biological process**	**9**
Anterior fat body protein	2
Conserved hypothetical protein AaeL_AAEL005270 (MF: transferase activity)	1
Phosphatidylethanolamine-binding protein (Immune response)	1
Cyclohex-1-ene-1-carboxyl-CoA hydratase (MF: catalytic activity)	1
Conserved hypothetical protein AaeL_AAEL015064 (MF: protein binding)	1
Lactoylglutathione lyase (MF: Lactoylglutathione lyase activity)	1
rho guanine dissociation factor (MF: Rho GDP-dissociation inhibitor activity)	1
Glutathione s-transferase (MF: protein binding)	1

^a^ Categories were taken from Gene Ontology annotations of biological process at VectorBaseDB. MF: Molecular function.

By comparing our data to the previously reported protein profiles of the midgut and midgut brush border membrane vesicles 2D maps of *Ae. aegypti*[14,23], we identified approximately 60% (25/40) and 47% (19/40) of the proteins, respectively, for the first time in the midgut 2D map of an *Aedes* spp. The presence of protein disulfide isomerase, chaperonin-60 and enolase among the most abundant proteins was in good agreement with the midgut proteome analysis of *Ae. aegypti*[14,23]. The congruence between the theoretical molecular mass and experimental MW of the identified proteins (Table 1) indicates that primarily full-length proteins were present in the midgut extract.

### Biological processes of the identified proteins and potential functional associations

**Figure 2:**
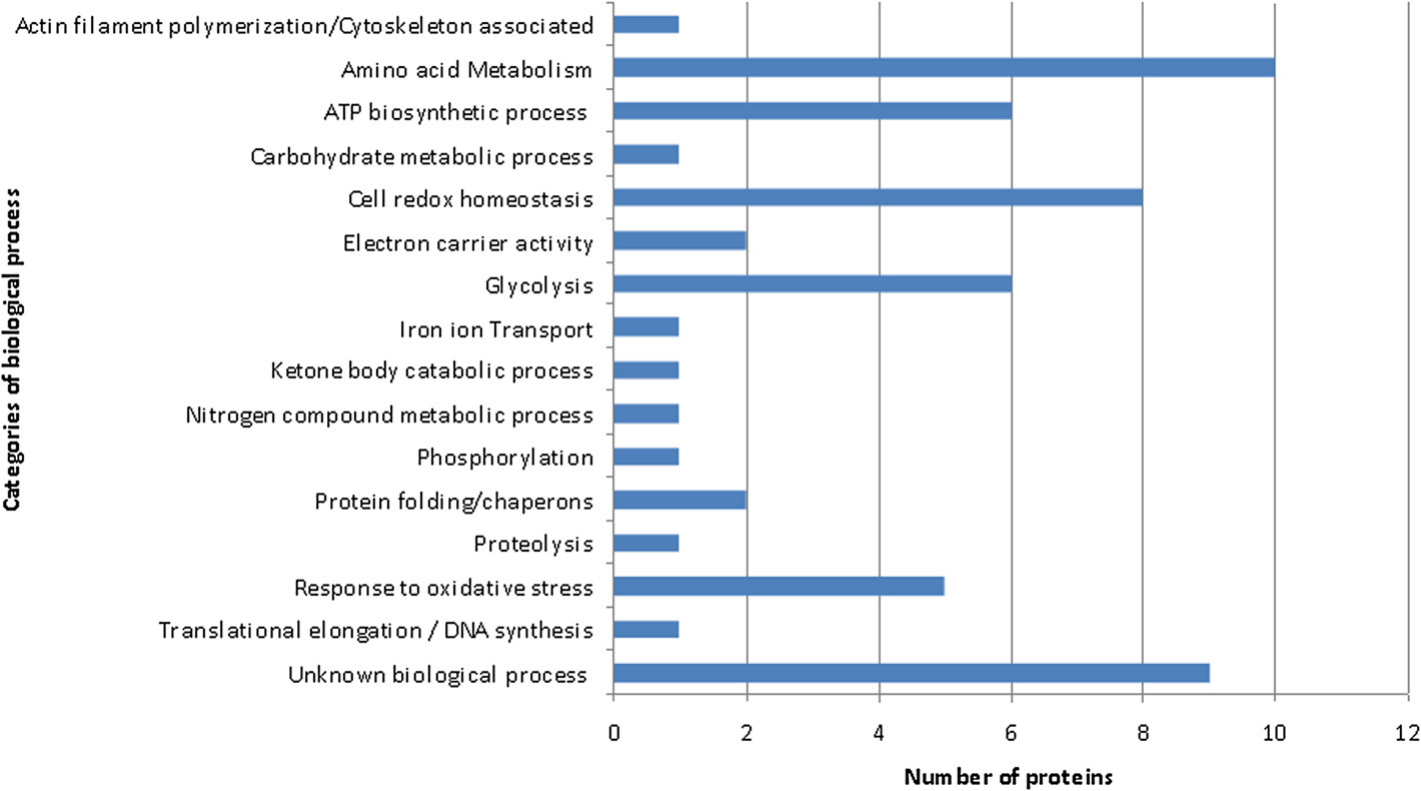
**Classification of the identified proteins in biological processes.** Categories were obtained from the gene ontology annotations of biological process at VectorBase DB.

*Ae. albopictus* proteins could be classified in 15 categories according to GO annotation of biological processes at the VectorBaseDB (Table 2, Figure 2). The most abundant groups correspond to proteins involved in amino acid metabolism, proteins with unknown biological process and proteins involved in the cell redox homeostasis (Figure 2). Other proteins were clustered into categories of those participating in the response to oxidative stress, (catalase and superoxide dismutase); phosphorylation (pyridoxine kinase); proteolysis (leucine aminopeptidase) and iron transport (transferrin), among others.

**Figure 3:**
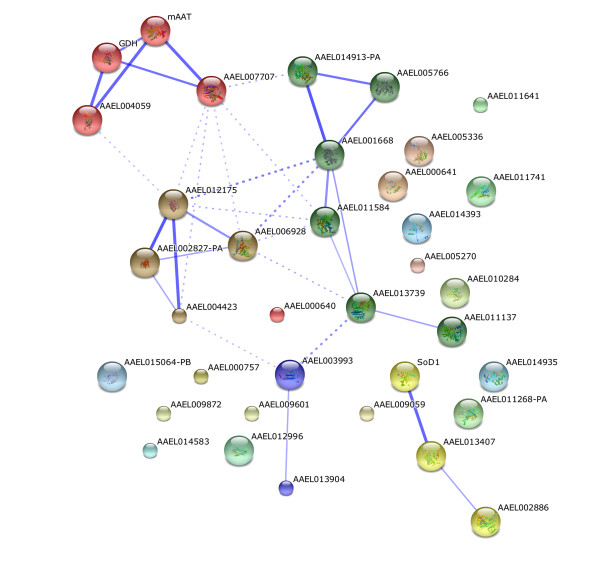
**Predicted functional associations among proteins identified in the midgut of sugar-fed *Ae. albopictus *females.** The proteins were analyzed using the STRING database 9.0. The predicted functional interaction networks are shown in the confidence view where the stronger associations are represented by thicker lines. The numbers correspond to the protein accession numbers in the VectorBase database and are described in Table 1.

Further information about the role of *Ae. albopictus* midgut proteins was obtained analyzing the potential functional associations among the identified proteins using the STRING 9.0 server, a database that provides information about known and predicted protein-protein interactions. This analysis revealed 5 consistent groups of functional associations (Figure 3). The first one is composed of proteins involved in glycolysis (pyruvate kinase, enolase, fructose-bisphosphate aldolase), protein folding (chaperonin-60, ch60), electron carrier (electron transport oxidoreductase), and ketone body catabolism (succinyl-CoA:3-ketoacid-coenzyme A transferase) (Figure 3, green cluster). The second functional association group includes proteins from carbohydrate and amino acid metabolism such as cystathionine beta-lyase, malate dehydrogenase, glutamate dehydrogenase and aspartate aminotransferase (Figure 3, red cluster). The repertoire of amino acid metabolism associated proteins identified in the midgut of the insects could be correlated to several factors: (i) constitutive expression of this group of proteins for the maintenance of basal metabolism, including protein turnover of midgut structural proteins; (ii) early after metamorphosis, females are fertilized and start to suffer changes in their metabolic machinery as preparation for the blood feeding; (iii) as midguts used in this study are from 2–5 day old females, they can present with proteins still remaining from the pupa. Such proteins would serve as substrate for metabolic activities detected, and (iv) given that before midgut dissection the microbiota was present in the midguts, bacteria and proteins produced by them could have induced the activation of insect midgut enzymes involved in amino acid metabolism.

In the third functional association network, the alpha and beta subunits of mitochondrial ATP synthase, dihydrolipoamide dehydrogenase and mitochondrial F0 ATP synthase D chain, which are functionally associated for ATP production, demonstrated consistent interactions (Figure 3, brown cluster). In addition, functional associations among these three groups were observed (Figure 3), revealing the complexity of the interactions required for the energetic metabolism of midgut cells.

The clusters indicated in green and brown both interact independently with a fourth group of functional association that includes the enzymes cyclohex-1-ene-1-carboxyl-CoA hydratase and 3-hydroxyisobutyrate dehydrogenase (Figure 3, blue cluster). Although the VectorBase DB did not assign the biological process in which the cyclohex-1-ene-1-carboxyl-CoA hydratase participates, the STRING analysis indicates that this enzyme could play a role in the interaction between glycolysis and the mitochondrial ATP production pathways. In our work, the enzymes cyclohex-1-ene-1-carboxyl-CoA hydratase and 3-hydroxyisobutyrate dehydrogenase were identified in the same spot (spot 18), and these two enzymes have also been identified together in the proteomic analysis of the midgut brush border membrane vesicles of *Ae. aegypti*[14], supporting the potential functional interaction of these enzymes. Finally, the enzyme cyclohex-1-ene-1-carboxyl-CoA hydratase has been recently proposed as part of the lipid metabolism pathway in *Ae. albopictus*[24].

The fifth network involves the functional interaction of superoxide dismutase, catalase and thioredoxin reductase, which interact in the detoxification of free radicals (Figure 3, yellow cluster). These enzymes play a key role in detoxification during carbohydrate meals, and it is expected that female midgut constitutively exhibits a substantial repertoire of these enzymes to deal with the intense oxidative stress induced during the digestion and absorption of a blood meal. In fact, it has been demonstrated that the expression of the genes coding for thioredoxin reductase and catalase in *Ae. aegypti* increase after blood feeding [25]. In addition, the expression of thioredoxin reductase and catalase in *Chironomus riparius* during stress by environmental pollutants, such as cadmium chloride, has been proposed as a biomarker of exposition to such contaminants [26,27]. All the other proteins identified in the 2D map appeared as isolated components in the functional association network (Figure 3).

**Figure 4:**
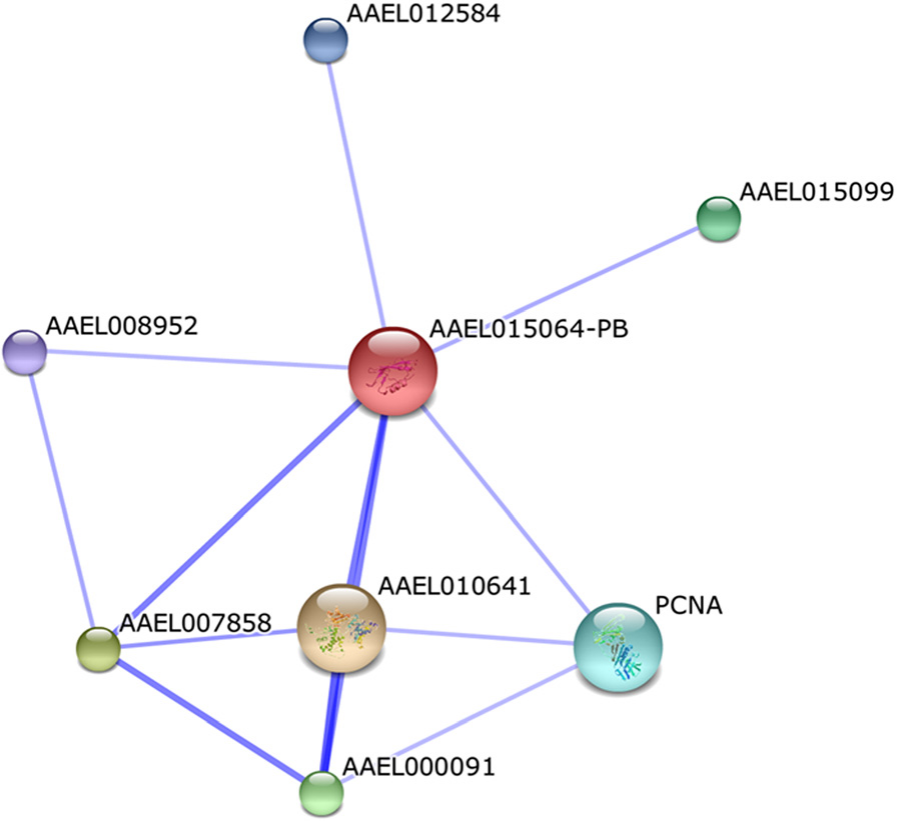
**Predicted functional interactions of the conserved hypothetical protein AAEL015064 as displayed by STRING.** The partners in this predicted interaction network potentially participate in the SUMO modification of proteins. The predicted functional interaction networks are shown in the confidence view where the stronger associations are represented by thicker lines. AAEL010641: Ubiquitin-activating enzyme E1; AAEL007858: ran GTPase-activating protein; AAEL000091: sumo-1-activating enzyme E1a; AAEL015099: sumo ligase; AAEL008952: sentrin/sumo-specific protease; PCNA: proliferating cell nuclear antigen; AAEL012584: DNA topoisomerase/gyrase.

The proteins classified under the “unknown biological process” category were individually submitted to functional association analysis in order to obtain insight into their potential function. No predicted associations were found for AAEL000757, AAEL005270, AAEL011268 and AAEL011741. On the other hand, for the conserved hypothetical protein AAEL015064, STRING predicted confident association with proteins involved in the potential SUMO modification of proteins, such as ubiquitin-activating enzyme E1 (AAEL010641), ran GTPase-activating protein (AAEL007858), sumo-1-activating enzyme E1a (AAEL000091), sumo ligase (AAEL015099), sentrin/sumo-specific protease (AAEL008952), proliferating cell nuclear antigen (PCNA) and DNA topoisomerase/gyrase (AAEL012584) (Figure 4). The SUMO pathway affects several cellular processes such as transport, apoptosis, and transcriptional regulation, among others [28]. Thus, in this work, we have identified a conserved protein that hypothetically plays a role in the SUMOylation pathway.

**Figure 5:**
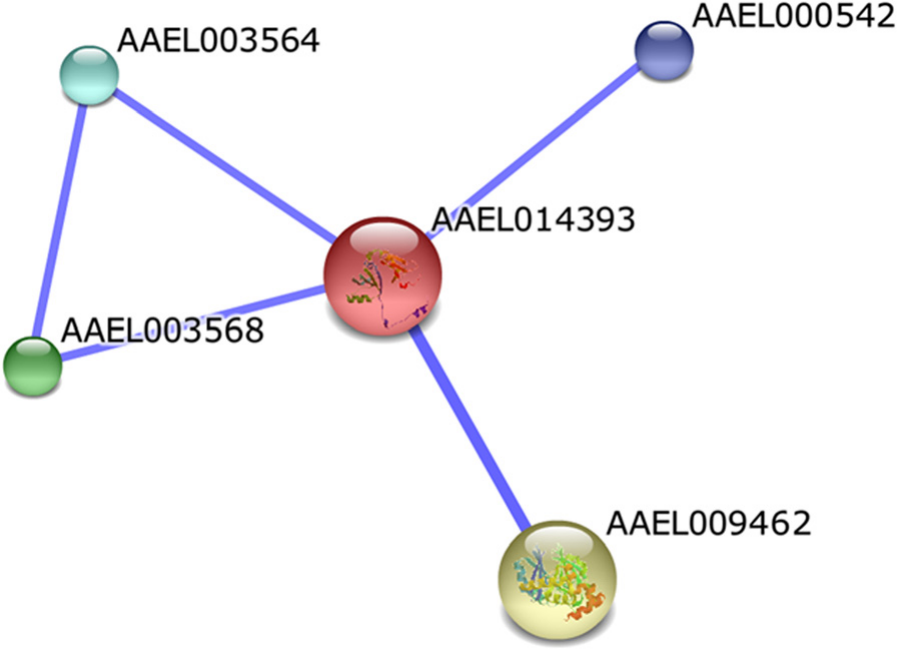
**Predicted functional interactions of the enzyme lactoylglutathione lyase (AAEL014393).** The proteins associated in this network participate in pyruvate metabolism. The predicted functional interaction networks are shown in the confidence view where the stronger associations are represented by thicker lines. AAEL009462: hydroxyacylglutathione hydrolase; AAEL003568: threonine dehydratase/deaminase; AAEL000542: conserved hypothetical protein.

The enzyme lactoylglutathione lyase (AAEL014393) exhibited predicted functional associations with hydroxyacylglutathione hydrolase (AAEL009462), threonine dehydratase/deaminase (AAEL003568) and with the conserved hypothetical protein (AAEL000542) (Figure 5). According to the KEGG, lactoylglutathione lyase and hydroxyacylglutathione hydrolase participate in the pyruvate metabolism pathway. In addition, lactoylglutathione lyase is involved in the metabolism of threonine.

**Figure 6:**
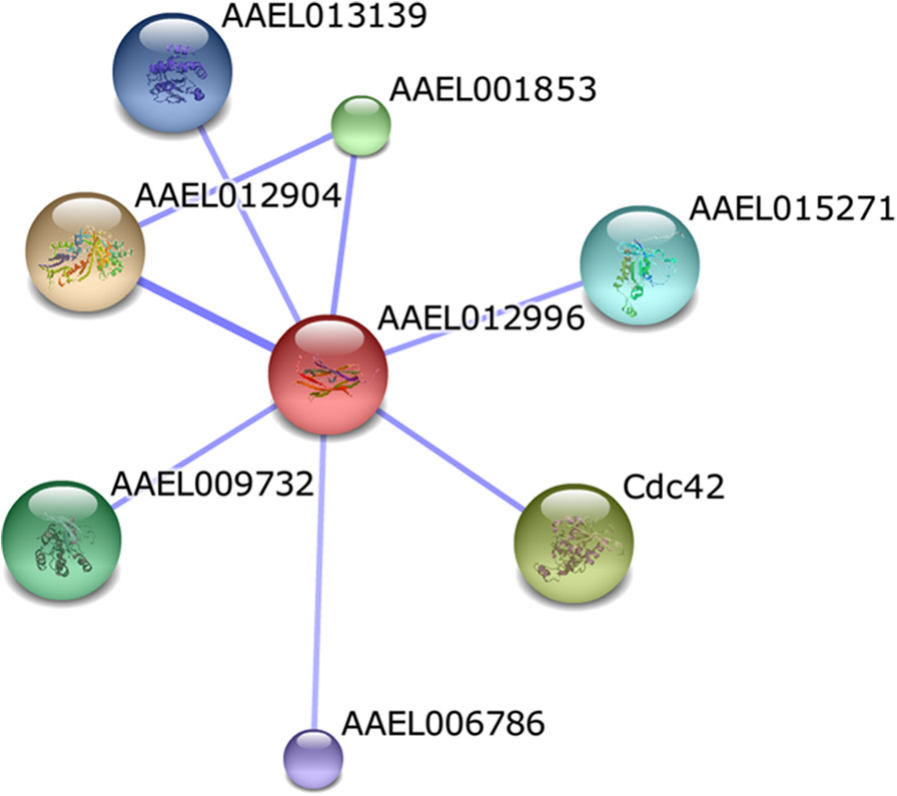
**Predicted functional interactions of the rho guanine dissociation factor (AAEL012996).** The proteins associated in this network may participate in various signaling pathways. The predicted functional interaction networks are shown in the confidence view where the stronger associations are represented by thicker lines. Cdc42, AAEL009732, AAEL015271: rac GTPases; AAEL012904: rab GDP-dissociation inhibitor; AAEL001853: rac-GTP binding protein; AAEL013139, AAEL006786: GTPase_rho.

The rho guanine dissociation factor (AAEL012996) exhibited predicted functional association with rac GTPases (Cdc42, AAEL009732, AAEL015271), rab GDP-dissociation inhibitor (AAEL012904), rac-GTP binding protein (AAEL001853) and GTPase_rho (AAEL013139, AAEL006786) (Figure 6). The GTPases of the Rho subfamily are involved in various signaling pathways that regulate the cell cycle progression and tissue morphogenesis [29,30]. It has also been demonstrated that rac GTPases participate in the *Drosophila* anti-parasitoid immune response [31].

Finally, although no predicted associations were found for phosphatidylethanolamine-binding protein (AAEL011268), this protein was shown to be associated with a protective effect against bacterial infection in *Drosophila*[32].

## Conclusion

In this study, we used 2DE combined with LC-MS/MS and data mining for mapping and identifying proteins expressed in the midgut of *Ae. albopictus* females fed exclusively on sugar. Analyses of subproteomes such as the one performed here, permit proteins with unknown function to be assigned to specific anatomical locations. In addition, data mining allowed us to assign potential functions to these proteins based on the functional association predictions. Our results also provided, for the first time, evidence on the expression and localization of proteins that were primarily assigned as hypothetical, thereby validating previous genome sequence predictions made in *Ae. aegypti*. This preliminary map of the *Ae. albopictus* midgut proteins will allow future comparisons of gene expression from the midgut of females fed with blood, making possible the identification of proteins that are exclusively expressed under a specific feeding condition.

## Competing interests

The authors declare that they have no competing interests.

## Authors’ contributions

JBJ, LSV and PC designed the study. LSV, ABV, CMR, and MJ performed the experimental work. LSV, PC and JBJ analyzed the data and prepared the manuscript with the critical input of MJ, GBD and CB. All authors read and approved the final manuscript.
